# Gallium-containing polymer brush film as efficient supported Lewis acid catalyst in a glass microreactor

**DOI:** 10.3762/bjoc.9.194

**Published:** 2013-08-16

**Authors:** Rajesh Munirathinam, Roberto Ricciardi, Richard J M Egberink, Jurriaan Huskens, Michael Holtkamp, Herbert Wormeester, Uwe Karst, Willem Verboom

**Affiliations:** 1 Laboratory of Molecular Nanofabrication, MESA+ Institute for Nanotechnology, University of Twente, P.O. Box 217, 7500 AE Enschede, The Netherlands; 2 University of Münster, Institute of Inorganic and Analytical Chemistry, Corrensstr. 28/30, 48149 Münster, Germany,; 3 Laboratory of Physics of Interfaces and Nanomaterials, MESA+ Institute for Nanotechnology, University of Twente, P.O. Box 217, 7500 AE Enschede, The Netherlands

**Keywords:** dehydration of oximes, flow chemistry, gallium, microreactors, Lewis acid catalysis, polymer brushes

## Abstract

Polystyrene sulfonate polymer brushes, grown on the interior of the microchannels in a microreactor, have been used for the anchoring of gallium as a Lewis acid catalyst. Initially, gallium-containing polymer brushes were grown on a flat silicon oxide surface and were characterized by FTIR, ellipsometry, and X-ray photoelectron spectroscopy (XPS). XPS revealed the presence of one gallium per 2–3 styrene sulfonate groups of the polymer brushes. The catalytic activity of the Lewis acid-functionalized brushes in a microreactor was demonstrated for the dehydration of oximes, using cinnamaldehyde oxime as a model substrate, and for the formation of oxazoles by ring closure of *ortho*-hydroxy oximes. The catalytic activity of the microreactor could be maintained by periodic reactivation by treatment with GaCl_3_.

## Introduction

Heterogeneous catalysis plays a crucial role in organic synthesis both in industry and academia. In the present situation, microreactors offer a number of benefits over classical setups [1–3]. Especially, heterogeneous catalysis in a continuous-flow microreactor is gaining growing attention, owing to its advantages such as increased surface-to-volume ratio, faster heat and mass transfer, only small amounts of reagents are handled, when compared to conventional laboratory equipment [4].

Heterogeneous catalysis in microreactors is carried out using two approaches, viz. a) with a packed-bed microreactor, where the catalyst is attached to a polymeric material enclosed in the microchannel [5], and b) when the catalyst is covalently connected to the inner walls of a glass microreactor [6]. Although the former approach has advantages such as high catalyst loading and easy fabrication of the catalytic device by filling the channels with functional catalytic particles, however, heat transfer limitations and pressure drop developing along the microchannel are serious drawbacks [7].

The literature contains numerous examples of Lewis acid catalysis [8–11]. However, only a limited number of papers are known dealing with heterogeneous Lewis acid catalysis, where a Lewis acid is tethered onto a solid surface like silica or gold [12–13]. Furthermore, to the best of our knowledge, there are no examples of the immobilization of a Lewis acid to a microreactor channel wall.

**Scheme 1:**
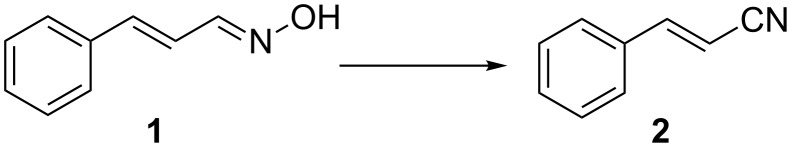
Gallium-catalyzed dehydration of cinnamaldehyde oxime (**1**).

Polymer brushes have proven to provide a unique platform in supported catalysis [14–15]. Previously, we have described the successful immobilization and evaluation of catalysts (e.g., basic organocatalyst [6], metallic nanoparticles [16], and enzymatic catalyst [17]) to the microchannel walls using polymer brushes. As a part of this program, herein, we report the anchoring of gallium as a Lewis acid catalyst making use of polystyrene sulfonate (PSS) polymer brushes. The choice for gallium was inspired by the successful application of solid supported gallium triflate to catalyze the Strecker reaction by Wiles and Watts [13]. The dehydration of cinnamaldehyde oxime [18] was used as a model reaction to study the catalytic activity of a microreactor with gallium immobilized onto its channel walls (Scheme 1).

## Results and Discussion

**Scheme 2:**
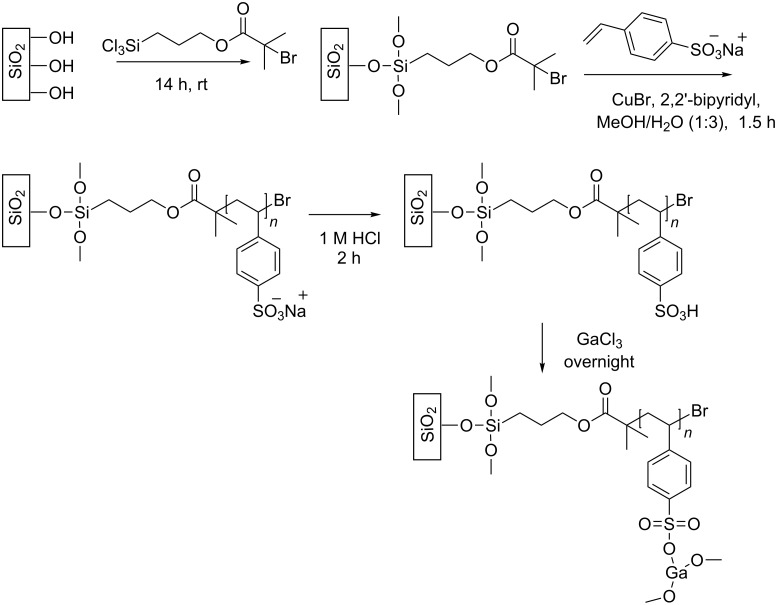
General scheme for anchoring of initiator, ATRP of styrene sulfonate, activation, and reaction with gallium chloride.

The catalytic polymer brush layer was first developed on a flat silicon oxide surface in order to optimize the reaction conditions before attempting the functionalization of a microreactor. Polystyrene sulfonate (PSS) polymer brushes [19] were synthesized according to the procedure summarized in Scheme 2. First, a monolayer of atom transfer radical polymerization (ATRP) initiator was covalently anchored on silicon oxide substrates [20]. Then, a solution of styrene sulfonate in methanol/water (1:3) in the presence of 2-2’-bipyridyl and CuBr, was used to grow the PSS polymer brushes by means of ATRP. After activation of the polymer brushes with 1 M HCl, they were incubated with a 100 mM solution of GaCl_3_ in acetonitrile.

Analysis of the surfaces by transmission FTIR spectroscopy after the polymerization step revealed the incorporation of sulfonic acid moieties, as it exhibited symmetric and asymmetric stretching vibrations at 1125 and 1011 cm^−1^, respectively [21]. The intensity of these peaks remained the same, after activation with HCl and upon treatment with GaCl_3_, indicating that the sulfonic acid moieties are still intact. Proof for the functionalization with gallium, however, was obtained by XPS spectroscopy. The atomic composition of the polymer brushes after anchoring of gallium was found to be C:O:S:Ga = 8:3.2:0.9:0.4, while no traces of chlorine were detected. From this, it was concluded that the polymer brushes contained on average one gallium per 2–3 styrene sulfonate groups. The thickness of the polymer brushes was determined by ellipsometry being about 77 nm in the dry state for a polymerization time of 1.5 h. Swelling studies on gallium-containing polymer brushes were performed in water, acetonitrile, and ethanol. Table 1 shows that the polymer brushes swell to the same extent in water as well as in organic solvents as acetonitrile and ethanol.

**Table 1:** Thickness of gallium-functionalized PSS polymer brushes on a flat silicon oxide surface.

	thickness (nm)

dry state	77 ± 2
water	91 ± 1
acetonitrile	95 ± 1
ethanol	96 ± 1

The same procedure used for the flat silicon oxide substrates was followed to immobilize gallium onto the interior of a glass microreactor with channel dimensions of 150 µm in width and depth, and having an internal volume of 13 µL. The PSS polymer brushes were grown and functionalized with gallium on the microchannel interior in the continuous flow-mode.

The dehydration of cinnamaldehyde oxime (**1**, 25 µM in acetonitrile) was used as a model reaction to study the catalytic activity of the gallium-functionalized microreactor, at 90 °C and 5 atm pressure, generated using a back pressure regulator in continuous flow. The conversion of the reagent was monitored online by in-line UV–vis detection, measuring the decrease in the extinction of the solution of oxime **1** at 286 nm. The reaction times were varied by changing the flow rates between 1 and 26 µL·min^−1^. The reaction showed nearly complete conversion at a residence time of 13 min. Under similar reaction conditions, after activating the PSS polymer brushes with 1 M HCl, no conversion was observed in the absence of gallium, proving that gallium is the catalytically active species. Under our conditions the reaction proceeded much faster when compared with the lab scale: the gallium(III) triflate (5 mol %) catalyzed dehydration of **1** in refluxing acetonitrile reported in literature [18] took 2 h to give **2** in 90% yield.

**Figure 1:**
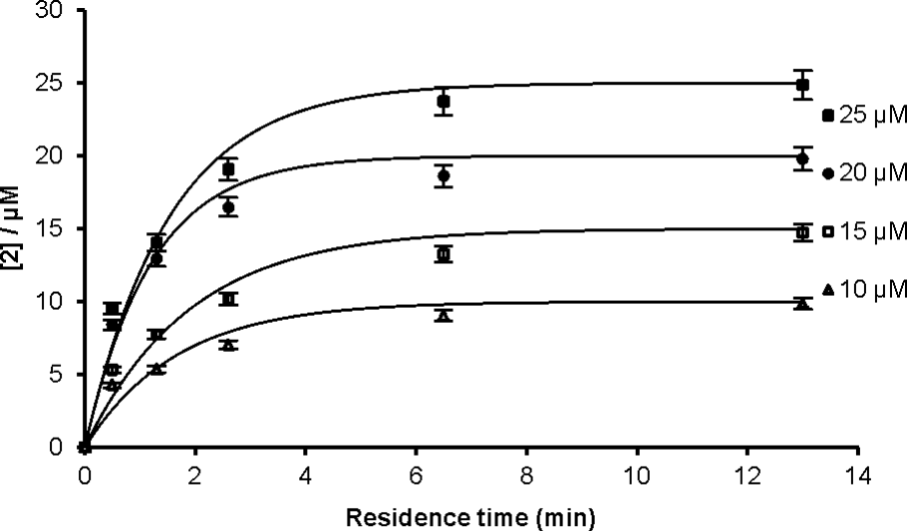
Gallium-catalyzed formation of nitrile **2** at 90 °C and 5 atm pressure.

A kinetic analysis of the dehydration of oxime **1** was performed by carrying out the reaction at different concentrations of **1** (10–25 µM, Figure 1). The experimental data were fitted to a first-order rate equation, giving an observed rate constant, *k*_obs_, of (11 ± 2) × 10^−3^ s^−1^. The values of the rate constants at different oxime concentrations were the same, within experimental error.

**Figure 2:**
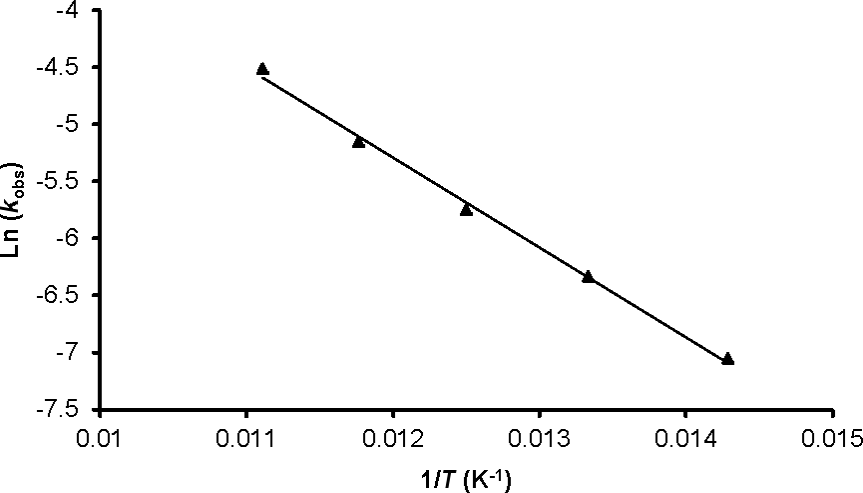
Arrhenius plot for the dehydration of cinnamaldehyde oxime (**1**).

The activation energy of the dehydration of oxime **1** was determined by calculating the *k*_obs_ values at different temperatures ranging from 70–90 °C (Supporting Information, Figure 1), with increments of five degrees. From the slope of the Arrhenius plot (Figure 2), the resulting activation energy was calculated to be 6.55 kJ·mol^−1^.

The substrate scope of the dehydration of oximes was extended using the same reaction conditions as above (Table 2). 4-Nitrobenzaldehyde oxime (**3**, Table 2, entry 1) resulted in a conversion of 62% within 13 min reaction time, while for the batch reaction, using gallium(III) triflate as a catalyst, 16 h at 120 °C was needed to give the nitrile **8** in 82% [18]. Anthracen-9-carbaldehyde oxime (**5**, Table 2, entry 3) showed a relatively poor conversion, which is ascribed to the steric hindrance of the molecule in reaching the catalytically active sites within the polymer brushes. In literature [18] this reaction, using gallium(III) triflate, required a reaction time of 8 h in refluxing acetonitrile to obtain the corresponding nitrile **10** in 87% yield. In case of 2-hydroxy-1-naphthaldehyde oxime (**6**) [22] (Table 2, entry 4) and salicylaldehyde oxime (**7**) [18] (Table 2, entry 5) dehydration happened between the *ortho*-hydroxy group and the oxime to give ring closure to the corresponding oxazoles **11** and **12**, respectively, in very good conversions.

**Table 2:** Dehydration of oximes in the gallium-functionalized catalytic microreactor^a^.

entry	substrate	product	conversion^b^

1 [18]	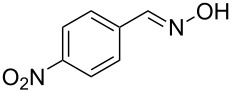 **3**	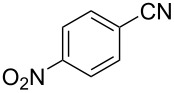 **8**	62
2 [23]	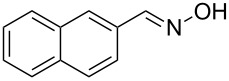 **4**	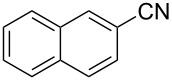 **9**	47
3 [18]	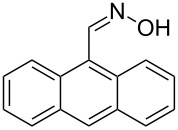 **5**	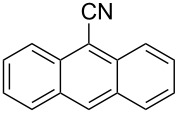 **10**	19
4 [22]	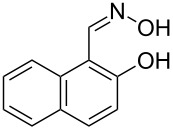 **6**	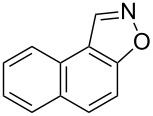 **11**	95
5 [18]	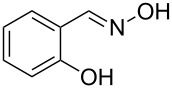 **7**	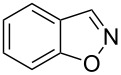 **12**	90

^a^All reactions were performed using 25 µM substrate in acetonitrile at 90 °C, 5 atm pressure, using a back pressure regulator, and a residence time of 13 min. ^b^Conversions were determined using online UV–vis spectroscopy by following the change in the extinction of the substrate at a specific wavelength.

**Figure 3:**
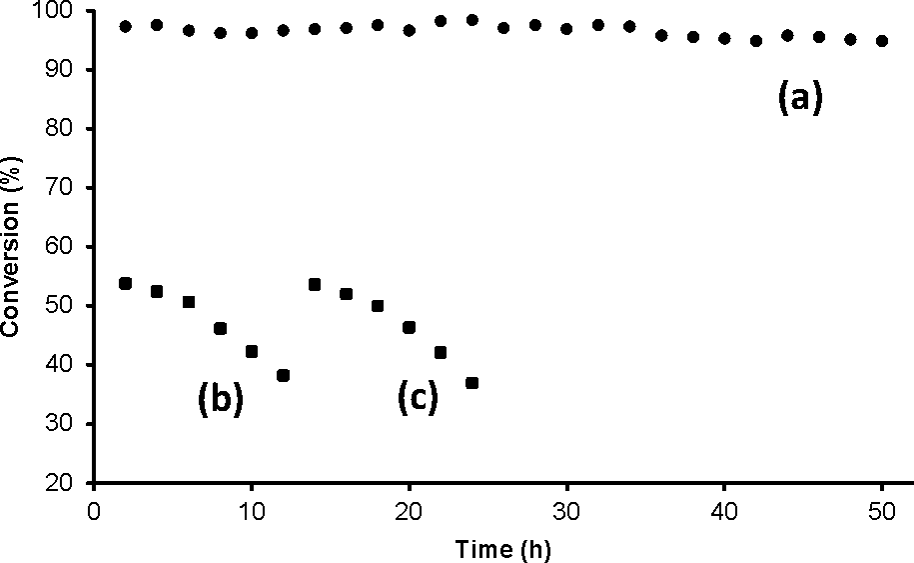
Conversion of cinnamaldehyde oxime (**1**, 25 µM in acetonitrile) by continuously running the catalytic microreactor: (a) for 50 h with 13 min residence time, (b) for 12 h with 78 s residence time, (c) also for 12 h with 78 s residence time but after reactivating the microreactor with GaCl_3_.

The dehydration of **1** (25 µM in acetonitrile), under similar conditions as mentioned above, was used to test the stability of the catalytic layer in the microreactor. When the catalytic microreactor was continuously run for 50 h with a residence time of 13 min (flow rate of 1 µL·min^−1^), the conversions remained nearly quantitative. However, with a shorter residence time of 78 s (flow rate of 10 µL·min^−1^), the conversions (maximum about 55%) gradually decreased. Total reflection X-ray fluorescence (TXRF) analysis of one of the samples with residence times of 78 s and 13 min, showed the presence of 1.0 ppm and 0.2 ppm of gallium, respectively. Nevertheless, the catalytic activity could be easily reactivated to the original value (error 2–3%) by treating the microreactor with 100 mM GaCl_3_ in acetonitrile overnight in the continuous flow mode (0.1 µL·min^−1^) (Figure 3). This proves that, at a flow rate of 1 µL·min^−1^, our catalytic system could be continuously used for at least two days without any noticeable decrease in the catalytic activity. If necessary, especially using higher flow rates, the catalytic system can be easily reactivated by treatment with GaCl_3_. To estimate the amount of gallium present in the polymer brushes, the catalytically active microreactor was deactivated by flowing 1 M HCl with a flow rate of 1 µL·min^−1^ and subsequent, thorough rinsing with water and acetonitrile. TXRF analysis of the solution showed the presence of 3.41 ± 0.01 µg of gallium. When the catalytic microreactor was activated and deactivated following the above mentioned protocol, the amount of gallium detected remained the same within the error limit.

## Conclusion

In conclusion, to the best of our knowledge, we described the first example of the application of polystyrene sulfonate-based polymer brushes to anchor gallium as a Lewis acid catalyst onto the microchannel interior of a microreactor and proved its catalytic activity for the dehydration of oximes to the corresponding nitriles and the ring closure of *ortho*-hydroxy oximes to the corresponding oxazoles. In general, our catalytic system showed faster conversions for most substrates than using lab scale conditions. XPS data, obtained on a flat silicon oxide surface, showed that on average one gallium per 2–3 styrene sulfonate groups of the PSS polymer brushes was incorporated. Upon slow deactivation the catalytic activity of the microreactor can be easily reactivated to its initial value by flowing through a solution of GaCl_3_. We believe that this approach has a wider scope and can be used to anchor other Lewis acids in a microreactor to study a range of Lewis acid-catalyzed reactions in an efficient way.

## Experimental

### Materials

The chemicals and solvents were purchased from Sigma-Aldrich unless otherwise stated and were used without purification unless specified. Single-side-polished silicon wafers were purchased from OKMETIC with (100) orientation. 3-(5’-Trichlorosilylpentyl) 2-bromo-2-methylpropionate was synthesized following a literature procedure [20]. CuBr was puriﬁed by washing with glacial acetic acid and, after ﬁltration, by rinsing with ethanol and acetone, was stored in a vacuum desiccator. Methanol and ethanol (VWR, analytical reagent grade) were used without further purification, water was purified with the Milli-Q pulse (MILLIPORE, R = 18.2 MΩ cm) ultra-pure water system, dry toluene was from an encapsulated solvent purification system (MB-SPS-800), and acetonitrile (for analysis EMSURE® ACS, Reag. Ph Eur, Merck).

### Methods

FTIR spectra were recorded using a Nicolet 6700 FTIR spectrometer. Ellipsometry measurements were performed with a Spectroscopic Ellipsometer M-2000X (J.A. Woolam Co., Inc.) with light reflected at 70° and a spot size of 2 mm diameter. Over a wavelength range of 340–1000 nm, with spectral resolution of about 2 nm both Psi and Delta were recorded as well as the intensity and amount of depolarization of the reflected light. The Complete EASE v.4.64 software package (J.A. Woolam Co., Inc.); was used to both control the instrument as well as for data analysis and modeling. For swelling experiments an in-situ homemade glass cell with an inner volume of about 70 mL was used. The 5 mm thick glass windows were positioned perpendicular to the light beam and were transparent in the employed wavelength range. For all measurements, the data were corrected for residual polarization by the windows. X-ray photoelectron spectroscopy (XPS) on the gallium-functionalized silicon oxide wafers were obtained on a Quantera Scanning X-ray Multiprobe instrument, equipped with a monochromatic Al Kα X-ray source producing approximately 25 W of X-ray power. XPS-data were collected from a surface area of 1000 μm × 300 μm with a pass energy of 224 eV and a step energy of 0.8 eV for survey scan and 0.4 for high resolution scans. For quantitative analysis, high resolution scans were used. Total reflection X-ray fluorescence analysis (TXRF) was carried out on a S2-PICOFOX instrument (Bruker AXS, Berlin, Germany) with an air-cooled molybdenum anode for X-ray generation. The excitation settings were 50 kV and 750 mA and quartz glass disks were used as sample carriers. The analysis was performed by signal integration over 500 seconds. For the determination, the signal of gallium (K_α1_ = 9.251 keV) was quantified by using strontium (K_α1_ = 9.251 keV) as internal standard ([Sr] = 10.0 µg/mL). Quantification was performed by the Bruker Spectra software (version 6.1.5.0) and based on the known concentration of the internal standard.

### Set up of the flow microreactor

All microreactor experiments were performed in a setup as described in reference [16]. A back pressure regulator (Future Chemistry) was connected to the outlet of the microreactor. Glass microreactors with a residual volume of 13 µL and dimensions of 150 µm depth and 150 µm width were purchased from Micronit Microfluidics (Enschede, The Netherlands).

### Synthesis of the catalytic polymer coating

Immobilization of the trichlorosilane initiator and the polymer brushes synthesis on the silicon oxide surface [19–20] and the microchannels [16] were carried out following literature procedures.

A solution of styrene sulfonate (1.25 g, 6.0 mmol) and 2,2’-bipyridyl (140 mg, 0.9 mmol) in a 3:1 mixture of methanol and water (12 mL) was degassed using the freeze-pump-thaw method (in a sealed Schlenk vessel). (The above-mentioned solution was frozen by immersion in liquid nitrogen. When the solvent was completely frozen the flask is kept under high vacuum for 5 min, with the flask still immersed in the liquid nitrogen. The flask was then closed and brought to room temperature until the solvent has completely melted. This process was repeated three times and after the last cycle the flask was filled with nitrogen.) CuBr (57 mg, 0.40 mmol) was added to this solution. To dissolve all solids, the mixture was stirred for 30 min under a continuous flow of nitrogen. Afterwards an initiator coated silicon wafer was placed in a Schlenk tube and the flask sealed with a septum. The tube was filled with argon and the monomer solution was syringed inside. For the polymerization in the device, the same solution was syringed through the microchannel till the device was completely filled. The solution was kept in contact with the silicon wafer and with the microchannel at a flow rate of 0.1 µL·min^−1^ for 1.5 h. After the polymerization, the silicon wafer and the microchannel were rinsed with water and methanol, and dried with a stream of nitrogen. In the next step the silicon wafers were soaked in a 1 M solution of HCl. The same solution was flowed with a flow rate of 0.1 µL·min^−1^ through the microreactor. After 2 h they were rinsed with water, acetone, and acetonitrile, and subsequently dried with a stream of nitrogen. For the preparation of the gallium-based Lewis acid bearing polymer brushes, all samples were first incubated overnight in a 100 mM solution of GaCl_3_ in acetonitrile. The same solutions were flowed at a flow rate of 0.1 µL·min^−1^ through the microreactor. Subsequently, the silicon wafer and microchannel were rinsed with acetonitrile.

### Kinetic study

The dehydration of cinnamaldehyde oxime (**1**, 10–25 µM) was carried out in acetonitrile at 90 °C under 5 atm pressure. Cinnamaldehyde oxime (**1**) and cinnamonitrile (**2**) exhibit the absorption maximum in acetonitrile at 286 nm and 271 nm, respectively. The conversion of cinnamaldehyde oxime (**1**) to cinnamonitrile (**2**) was calculated based on the decrease in the absorption at 286 nm using the formula: [**2**] = (ε_286_ (**1**) · [**1**]_0_ − *A*_observed_) / (ε_286_ (**1**) − ε_286_ (**2**)) (*A*_observed_ = absorbance measured experimentally, while carrying out the reaction). The molar absorptivities (ε) of cinnamaldehyde oxime (**1**) and cinnamonitrile (**2**) in acetonitrile at 286 nm are 35640 and 17800 L·mol^−1^·cm^−1^, respectively. The *k*_obs_ values were calculated by fitting the experimental data with the following equation: [**2**] = [**1**]_0_ · (1 − exp (−*k*_obs_·*t*)) using least-squares fit. The experimental errors in these measurements are ±4%.

### Substrate scope

The dehydration reaction with different oxime substrates (Table 2, entries 1–5) was carried out in a catalytic microreactor at 90 °C, 5 atm pressure, with a residence time of 13 min. A substrate concentration of 25 µM was used for all the substrates. The conversions were determined using online UV–vis spectroscopy by following the change in the extinction of a substrate specific wavelength. In case of 2-hydroxy-1-naphthaldehyde oxime (**6**, Table 2, entry 4) and salicylaldehyde oxime (**7**, Table 2, entry 5), the conversions were determined by following the decrease in the absorbance at 353 nm (ε_353_ = 8360 L·mol^−1^·cm^−1^) and 310 nm (ε_310_ = 4820 L·mol^−1^·cm^−1^), respectively; the corresponding oxazoles **11** and **12** showed no absorbance in that region. With 4-nitrobenzaldehyde oxime (**3**, Table 2, entry 1) and 2-naphthaldehyde oxime (**4**, Table 2, entry 2), the conversions were determined by following the decrease in the absorbance at 303 nm (ε_303_ = 20560 L·mol^−1^·cm^−1^) and 283 nm (ε_283_ = 20360 L·mol^−1^·cm^−1^), respectively, using the formula that was applied for **1**, as the corresponding nitriles **8** and **9** showed molar absorptivities (ε) of 2200 and 7160 L·mol^−1^·cm^−1^, respectively, in that region. In case of anthracen-9-carbaldehyde oxime (**5**, Table 2, entry 3), the conversions were determined by following the increase in the absorbance at 403 nm (ε_403_ = 2600 L·mol^−1^·cm^−1^); the corresponding product **10** has a molar absorptivity (ε) of 13400 L·mol^−1^·cm^−1^ in that region.

### On-line UV–vis detection

The conversion of the oximes was followed using online UV–vis spectrometry as described in reference [16].

## Supporting Information

File 1 Conversion of cinnamaldehyde oxime (**1**, 25 µM in acetonitrile) catalyzed by gallium in a microreactor at different temperatures.
