# Supplementary material for: Gallium-containing polymer brush film as efficient supported Lewis acid catalyst in a glass microreactor
Source: Beilstein J Org Chem. 2013 Aug 16;9:1698–704. doi: 10.3762/bjoc.9.194 (PMC3778416; doi:10.3762/bjoc.9.194)
Supplement: File 1 — Conversion of cinnamaldehyde oxime (1, 25 µM in acetonitrile) catalyzed by gallium in a microreactor at different temperatures. [file Beilstein_J_Org_Chem-09-1698-s001.pdf]

Supporting information

for

Gallium-containing polymer brush film as efficient supported Lewis acid catalyst in a glass microreactor

Rajesh Munirathinam[1], Roberto Ricciardi[1], Richard J. M. Egberink[1], Jurriaan Huskens[1], Michael Holtkamp[2], Herbert Wormeester[3], Uwe Karst[2] and Willem Verboom*[1]

Address: [1]Laboratory of Molecular Nanofabrication, MESA+ Institute for Nanotechnology, University of Twente, P.O. Box 217, 7500 AE Enschede, The Netherlands, [2]University of Münster, Institute of Inorganic and Analytical Chemistry, Corrensstr. 28/30, 48149 Münster, Germany, and [3]Laboratory of Physics of Interfaces and Nanomaterials, MESA+ Institute for Nanotechnology, University of Twente, P.O. Box 217, 7500 AE Enschede, The Netherlands.

Email: Willem Verboom* - w.verboom@utwente.nl

* Corresponding author

Conversion of cinnamaldehyde oxime (1, 25 µM in acetonitrile) catalyzed by gallium in a microreactor at different temperatures.

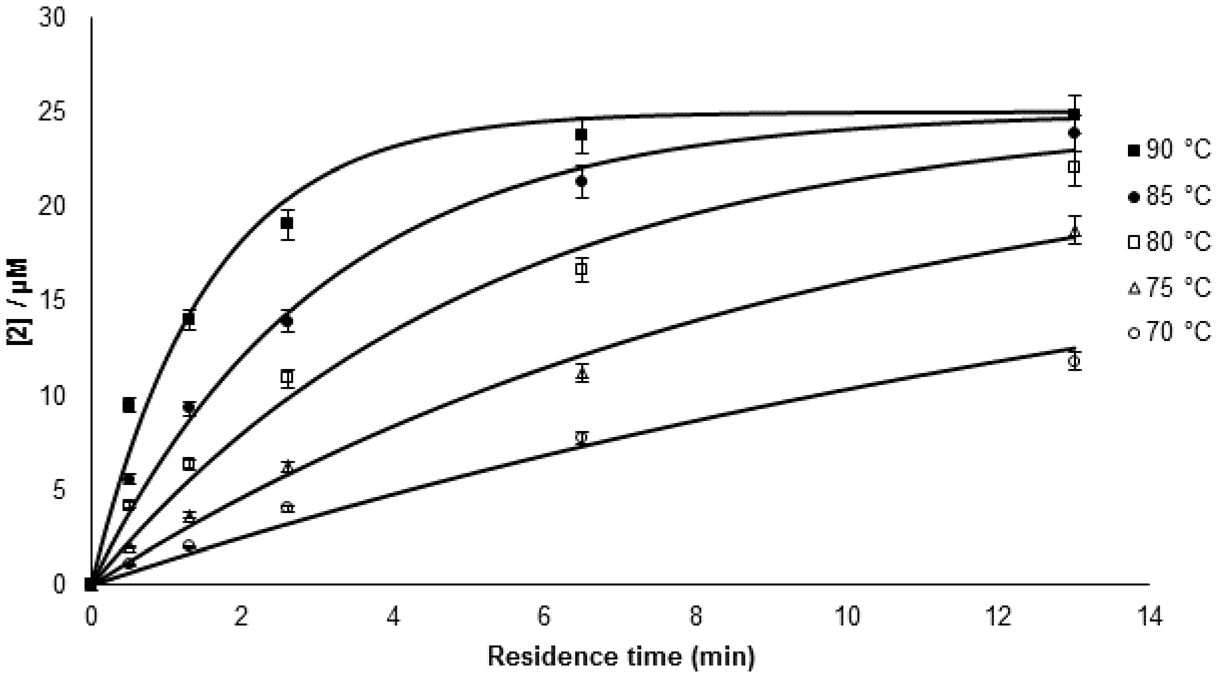

Figure 1: Conversion of cinnamaldehyde oxime (**1**, 25 µM in acetonitrile) catalyzed by gallium in a microreactor at different temperatures.
